# Occupancy of the Invasive Feral Cat Varies with Habitat Complexity

**DOI:** 10.1371/journal.pone.0152520

**Published:** 2016-09-21

**Authors:** Rosemary Hohnen, Katherine Tuft, Hugh W. McGregor, Sarah Legge, Ian J. Radford, Christopher N. Johnson

**Affiliations:** 1 School of Biological Sciences, University of Tasmania, Hobart, Tas, 7001, Australia; 2 Australian Wildlife Conservancy, Mornington Wildlife Sanctuary, PMB 925, Derby, WA, 6728, Australia; 3 Threatened Species Recovery Hub, National Environmental Science Program, Charles Darwin University, NT, 0900, Australia; 4 Western Australian Government, Department of Parks and Wildlife, PO Box 942, Kununurra, WA, 6743, Australia; University of California-Berkeley, UNITED STATES

## Abstract

The domestic cat (*Felis catus*) is an invasive exotic in many locations around the world and is thought to be a key factor driving recent mammal declines across northern Australia. Many mammal species native to this region now persist only in areas with high topographic complexity, provided by features such as gorges or escarpments. Do mammals persist in these habitats because cats occupy them less, or despite high cat occupancy? We show that occupancy of feral cats was lower in mammal-rich habitats of high topographic complexity. These results support the idea that predation pressure by feral cats is a factor contributing to the collapse of mammal communities across northern Australia. Managing impacts of feral cats is a global conservation challenge. Conservation actions such as choosing sites for small mammal reintroductions may be more successful if variation in cat occupancy with landscape features is taken into account.

## Introduction

The free-ranging domestic cat (*Felis catus*) is an adaptable predator and as an invasive species is a threat to biodiversity on a global scale [1–3]. Feral cats can have devastating impacts on vulnerable prey taxa and are responsible for over 14% of modern bird, mammal, and reptile extinctions [1, 4]. Impacts of feral cats are particularly high in systems where the native fauna lacks previous exposure to cats or analogous predators, and therefore exhibit few morphological and behavioural defences against cat predation, competition with cats, and cat-borne diseases [1, 5, 6]. Identifying other factors that influence and potentially mitigate the impacts of cat predation is a global conservation priority [2, 7, 8].

Predation by feral cats is thought to be primarily responsible for recent declines of small mammal populations across northern Australia [9–11]. Declines have been especially severe for species that live in open and topographically simple habitats such as savanna or grassland [7, 12]. In contrast, species that inhabit dense vegetation such as rainforest or that live in areas with high topographic complexity have declined least [7, 12, 13]. These patterns are consistent with the broader continent-wide pattern of mammal decline and persistence in Australia [9, 14–16].

The persistence of small mammals in complex habitats could indicate a large-scale habitat preference by cats whereby cats occupy complex habitats less. On the other hand, prey might persist in such habitats despite the presence of cats, due to availability of refuges that allow them to avoid predation, or for other reasons. We tested these ideas in north-western Australia by measuring cat occupancy and mammal abundance in habitats with contrasting topographic complexity. Our results show that cats are rare in structurally complex habitats where small mammals remain most abundant, suggesting that low occupancy of cats in such habitats accounts for their importance as refuges for small mammals.

## Methods

### Study area

**Fig 1:**
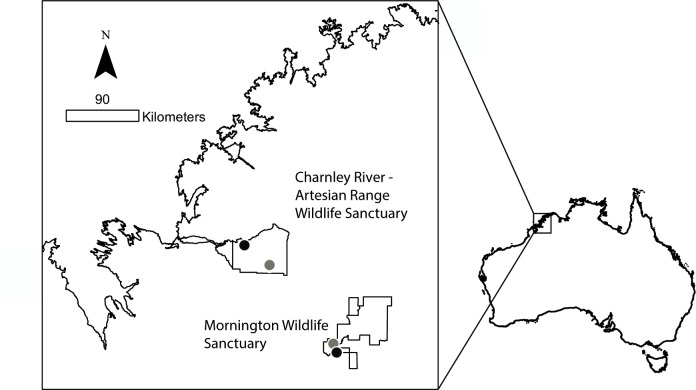
Locations of camera arrays in topographically complex (black circles) and simple (grey circles) habitats, in the north and central Kimberley, north-western Australia.

The study was conducted on Mornington Wildlife Sanctuary (17°30’S, 126°06’E, 320, 000 ha) and Charnley River-Artesian Range Wildlife Sanctuary (16°24’S, 125° 30’E, 172, 738 ha), both properties managed by the Australian Wildlife Conservancy (AWC) in the Kimberley region of Western Australia (Fig 1). The region has a tropical monsoonal climate with three main seasons, the wet (December-March), the early dry (April-July) and the late dry (August-November). Annual rainfall averages over 750 mm on Mornington Wildlife Sanctuary and over 1100 mm on Charnley River-Artesian Range Wildlife Sanctuary [17]. Vegetation of the study areas is dominated by eucalypt woodland, with a grass layer composed of both perennial (*Triodia* spp., *Dicanthium* spp., *Aristida* spp., *Chrysopogon fallax*, *Sehima nervosum*, *Themeda traiandra*) and annual species (*Sorghum stipodeum*).

### Camera deployment

**Fig 2:**
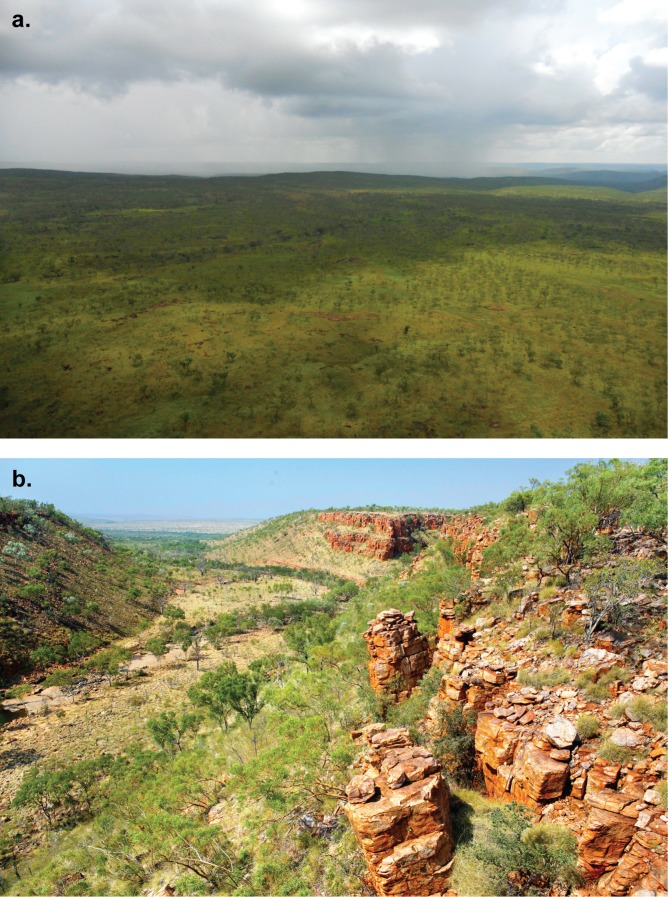
Photos of topographically simple (a.) and complex (b.) habitats in the Kimberley, Western Australia (photo credits: Wayne Lawler/AWC).

Four remote camera arrays were deployed between August 2011 and May 2014 (S1 Table), two on Mornington Wildlife Sanctuary in the central Kimberley and two on Charnley River-Artesian Range Wildlife Sanctuary in the north Kimberley (Fig 1). At each sanctuary, one array was deployed in a topographically complex rocky range and another in adjacent open plains. Topographically complex habitats were defined as landscapes where rock complexity was available, in the form of scree, cliffs and rock outcrops, and where elevation varied sometimes a much as 200 m across the array. Topographically simple habitats were defined as open plains with no rock structures available, and minimal elevation change (Fig 2).

Each array consisted of between 14 and 21 cameras (Reconyx Rapidfire Professional cameras 600c, Holmen, Wisconsin, USA), deployed for between 22 and 37 days. Cameras were placed on dry creek beds between 800 m– 1 km apart to decrease the likelihood that several cameras would be placed within an individual cat’s home range. Feral cats in the north Kimberley display strong territoriality with minimal overlap between home ranges [18, 19]. Cameras were set 30–40 cm off the ground, and programmed to take three images per trigger, one second apart, with no minimum time delay between triggers. All cameras were baited with 50 g of soil containing the urine from a single female sterilised cat, placed approximately 40 cm in front of the camera. Obstructions in front of the cameras were removed and cameras in topographically complex and simple habitats had the same field of view of between 0 to 30.5 m away.

### Occupancy analysis

We used occupancy modelling to examine how location and habitat complexity related to cat occupancy in the Kimberley landscape. Single-species occupancy models were used as we assumed that occupancy of cats at a population scale did not change during each camera deployment period. A site was defined as a single camera within an array, and a visit was defined as a sighting of a cat within a given 24-hour period. In each model the response variable was defined as the detection history of all cameras, where 1 indicated the presence of a cat, and 0 indicated absence of a cat, during a given 24 hour period. Probability of occupancy was modelled as a function of two camera site covariates: location (north or central Kimberley) and habitat complexity (topographically complex or simple). In all models except the null model, the probability of detection was modelled as a function of lure effectiveness, which was expected to decline at a constant rate during the study period. Other variables such as location and habitat complexity were not expected to effect detectability. We fitted occupancy models using the *unmarked* package in the program R version 3.03 [20, 21]. Models were compared using AIC scores and weights, with the models within two ΔAIC of the top model considered competitive.

### Small mammal relative abundance

Relative abundance of small mammals was estimated from AWC fauna monitoring data. For each array location, the total number of individuals captured in cage and Elliot traps, and the number of trap nights, was pooled across monitoring sites situated both within 20 km, and within the respective habitat, of each camera array (Table 1). Trap success was calculated by dividing the number of individuals by the number of trap nights.

**Table 1:** Measures of mammalian trap success pooled across fauna monitoring sites from around camera array locations in the north and central Kimberley, north-western Australia.

Location	Topography	Year	Sites	Trap nights	Individuals	Trap success
North Kimberley	complex	2013	5	1352	197	0.146
	simple	2014	9	810	16	0.020
Central Kimberley	complex	2006	11	2301	432	0.188
	simple	2012	48	4320	182	0.042

#### Ethical statement

All field methods used in this study were approved by the University of Tasmania Animal Ethics Committee (A12516), and the Western Australian Department of Parks and Wildlife Animal Ethics Committee (DPaW 2013/37 and AEC 2013/24). Field research was conducted with permission on Mornington Wildlife Sanctuary, and Charnley River-Artesian Range Wildlife Sanctuary, owned and managed by the Australian Wildlife Conservancy, ph: +61 8 9191 7014. We confirm that the field studies did not involve endangered or protected species.

## Results

### Detections

We detected feral cats on 74 of 1695 total trap-nights (S2 Table). Cats were not detected in topographically complex habitats of the north Kimberley, but in the central Kimberley cats were detected on 7 occasions. In contrast cats were detected in topographically simple habitats of the north Kimberley on 14 occasions and on 53 occasions in the central Kimberley.

### Occupancy models

**Fig 3:**
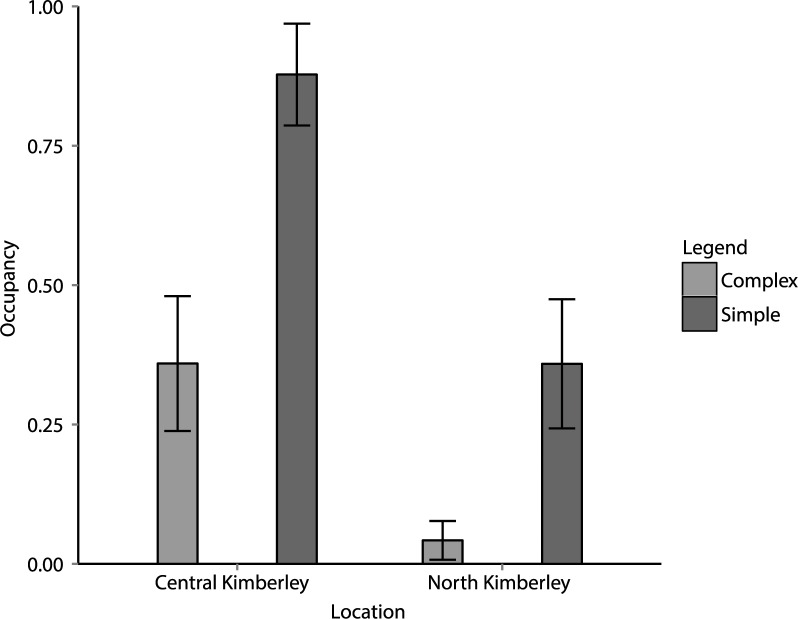
Feral cat occupancy estimates in topographically simple and complex habitats in the central and north Kimberley, north-western Australia.

The top-ranking model that best described occupancy of cats included the variables location (north or central) and topographic complexity (complex or simple), and had a model weight of 0.9. No other models were within two ΔAIC of this model (Table 2). Variable coefficients of the top model indicated that occupancy was higher in topographically simple habitats than complex habitats, and higher in the central Kimberley than in the north Kimberley (Table 3). Feral cat occupancy was also higher in the central Kimberley than the north Kimberley (Fig 3).

**Table 2:** Candidate models of feral cat occupancy in open and complex habitats of the north and central Kimberley, north-western Australia.

Model	K	AIC	ΔAIC	*w*
Ψ (Complexity + Location), p(lure)	5	565.29	0	0.90
Ψ (Location), p(lure)	4	571.15	5.85	0.05
Ψ (Complexity), p(lure)	4	571.62	6.33	0.04
Ψ (.), p(lure)	3	573.50	8.20	0.02
Ψ (.), p (.)	2	589.36	24.07	0.00

**Table 3:** Model specific coefficient estimates of feral cat occupancy in topographically simple and complex habitats in the north and central Kimberley, north-western Australia (where βCo = complex, βo = simple, and where βLn = north Kimberley, βo = central Kimberley).

Model	β₀ estimate ±se	βCo estimate ±se	βLn estimate ±se
Ψ (.), p(.)	-0.416±0.254	-	-
Ψ (.), p(lure)	0.116±0.307	-	-
Ψ (Complexity), p(lure)	-0.739±0.521	1.322±0.656	-
Ψ (Location), p(lure)	0.577±0.402	-	-1.181±0.612
Ψ (Complexity + Location), p(lure)	-0.381±0.538	2.153±0.881	-2.058±0.862

#### Small mammal trap success

Trap success was highest at the topographically complex sites in the central (0.188) and north Kimberley (0.146), followed by topographically simple sites in the central (0.042), and north Kimberley (0.020) (Table 1).

## Discussion

In both the north and central Kimberley, feral cats occupied topographically complex habitats considerably less than adjacent open plains. Diversity and abundance of mammals is high in topographically complex regions of the north Kimberley, and for many small mammal species this is one of the last places they persist on mainland Australia [7]. The lower occupancy of cats in these habitats of the north Kimberley may contribute to higher abundance and diversity of resident small mammal populations. Potentially this pattern may be driven by the lower hunting success of feral cats in rocky or topographically complex landscapes [22].

Across Australia the impacts of feral cats on mammals have been considered most detrimental where understory vegetation is sparse [23, 24], such as in arid systems [25, 26]. In north-western Australia feral cats preferentially hunt in areas where the understorey has been simplified, such as habitats that have been grazed or burnt by intense fires [19]. In these habitats feral cats were found to have greater hunting success when compared to grassy or rocky habitats [22]. In New Zealand, the avoidance of topographically complex areas by feral cats was also thought to be related the difficulty of moving across and catching prey in rugged habitats [27].Variation in hunting success between topographically complex and simple habitats may contribute to differences in cat occupancy observed in this study.

Variation in dingo density has also been suggested as a mechanism that determines spatial variation in the impact of cats between regions of the Kimberley [28]. Dingoes were detected on some cameras, but to test the relationship between dingoes, cats and topography, the deployment of replicate arrays with an appropriate distance between cameras (to maintain camera site independence for the analysis of dingo occupancy) would be required.

As each array was deployed on a separate year there is potential that differences in occupancy detected between sites in this study, reflect annual variation in population size rather than habitat variables. However this appears unlikely as monitoring of cat populations across multiple years at Mornington Wildlife Sanctuary in the central Kimberley, has not detected large fluctuations in feral cat population size [18]. This suggests differences in occupancy detected in this study are more likely to be driven by habitat.

Managing impacts of feral cats is a conservation priority across the world [2, 7, 8]. The results of this study suggest that topographically complex areas may offer small mammal communities some degree of refuge from predation by feral cats. Targeted management (including reintroductions) of small mammal populations may be more successful in areas where cover such as rock complexity is available. Targeted cat management around the fringe of topographically complex areas may also assist in further limiting cat impacts in these habitats. This study adds to a body of research that highlights the importance of understanding how invasive predator populations vary with landscape features [27], so that natural resilience can be acknowledged and used to inform more effective conservation and management strategies.

## Supporting Information

S1 Table Dates of camera deployment for each array. (DOCX)

S2 Table Presence and absence of feral cats at camera sites in the north and central Kimberley in complex and open landscapes (1 = present, 0 = absent). (CSV)
