# Supplementary material for: Occupancy of the Invasive Feral Cat Varies with Habitat Complexity
Source: PLoS One. 2016 Sep 21;11(9):e0152520. doi: 10.1371/journal.pone.0152520 (PMC5031312; doi:10.1371/journal.pone.0152520)
Supplement: S1 Table — (DOCX) [file pone.0152520.s001.docx]

**S1 Table: Dates of camera deployment for each array.**

| Location | Topography | Dates | Days |
| --- | --- | --- | --- |
| North Kimberley | complex | 2/11/13-8/12/13 | 37 |
|  | simple | 13/4/14-6/5/14 | 24 |
| Central Kimberley | complex | 4/8/11-7/9/11 | 35 |
|  | simple | 18/9/12-18/10/12 | 31 |
